# Development of a mitochondria-related gene signature for prognostic assessment in diffuse large B cell lymphoma

**DOI:** 10.3389/fonc.2025.1542829

**Published:** 2025-03-20

**Authors:** Fujue Wang, Yu Gao, Yue Chen, Pian Li, Yao Zeng, Yingying Chen, Yangcui Yin, Yongqian Jia, Yongsheng Wang

**Affiliations:** ^1^ State Key Laboratory of Biotherapy, Cancer Center, West China Hospital, Sichuan University, Chengdu, China; ^2^ Department of Hematology, The First Affiliated Hospital, Hengyang Medical School, University of South China, Hengyang, China; ^3^ Division of Thoracic Tumor Multimodality Treatment, Cancer Center, West China Hospital, Sichuan University, Chengdu, China; ^4^ Clinical Trial Center, West China Hospital, Sichuan University, Chengdu, China; ^5^ Department of Radiation Oncology, The First Affiliated Hospital of Guangxi Medical University; Guangxi tumor radiation therapy clinical medical research center, Nanning, China; ^6^ Department of Medical Oncology, Sichuan Clinical Research Center for Cancer, Sichuan Cancer Hospital & Institute, Sichuan Cancer Center, University of Electronic Science and Technology of China, Chengdu, China; ^7^ Department of Hematology, West China Hospital, Sichuan University, Chengdu, China

**Keywords:** diffuse large b cell lymphoma (DLBCL), mitochondrial-related genes (MitoRGs), prognosis, international prognostic index (IPI), phosphoenolpyruvate carboxykinase 2 (PCK2)

## Abstract

**Background:**

Mitochondria-related genes (MitoRGs) play a critical role in the pathogenesis of various cancer types. This study aims to develop a novel prognostic model based on a MitoRGs signature for patients with diffuse large B cell lymphoma (DLBCL).

**Methods:**

Clinical data and gene expression profiles of DLBCL patients were obtained from four datasets in the Gene Expression Omnibus (GEO) database. The Least Absolute Shrinkage and Selection Operator (Lasso) Cox regression analysis, along with multivariate Cox regression analysis, was employed to develop a prognostic MitoRGs signature for patients with DLBCL within the training cohort. The prognostic efficacy of the model was assessed using Kaplan-Meier survival analysis and Receiver Operating Characteristic (ROC) curve analysis. The validation cohorts were used to substantiate the model’s predictive capability. Single-sample gene set enrichment analysis (ssGSEA) was employed to examine immune infiltration across various risk groups, and the sensitivities to potential therapeutic agents for patients with DLBCL were also assessed. The role of the mitochondrial-related gene PCK2 in cell proliferation and apoptosis was investigated under varying glucose concentrations.

**Results:**

An eight-MitoRG signature exhibited independent prognostic significance and robust predictive capability for the survival outcomes of DLBCL patients. Notably, it effectively predicted prognosis across various DLBCL patient subgroups and enhanced the prognostic utility of the International Prognostic Index (IPI) score. Analyses utilizing ssGSEA and assessments of drug sensitivities identified distinct patterns of immune infiltration and differential responses to therapeutic agents among patients stratified into various risk groups. Moreover, a prognostic nomogram integrating age, IPI score, and MitoRGs signature was further developed, demonstrating enhanced prognostic accuracy and clinical applicability for DLBCL patients. In addition, research on phosphoenolpyruvate carboxykinase 2 (PCK2) indicated that silencing PCK2 expression inhibits cellular proliferation and induces apoptosis under conditions of low glucose.

**Conclusion:**

We developed an innovative prognostic MitoRGs signature to predict outcomes and enhance the prognostic utility of the IPI score in DLBCL, offering a novel perspective for the treatment of DLBCL.

## Introduction

Diffuse large B-cell lymphoma (DLBCL) is the most prevalent form of non-Hodgkin lymphoma (NHL), with around 150,000 new cases diagnosed annually (1). More than 60% of DLBCL patients receive standard treatment, typically the R-CHOP regimen, which combines rituximab, cyclophosphamide, doxorubicin, vincristine, and prednisone, achieving long-term remission. However, nearly one-third of DLBCL patients experience refractory or relapse (R/R) due to the significant heterogeneity of DLBCL (2, 3). Recently, many new therapies have emerged for DLBCL patients, particularly those with R/R. These include CD19-directed chimeric antigen receptor T-cell (CD19 CAR-T) and bispecific antibodies (BsAbs), but their broader application is restricted by issues such as accessibility, high costs and toxicity-related adverse events (AEs) (4).

Clinically, common prognostic evaluation systems like the International Prognostic Index (IPI), revised IPI (R-IPI) and the National Comprehensive Cancer Network-IPI (NCCN-IPI) rely on clinical variables for predicting the prognosis in DLBCL patients due to their simplicity. However, they overlook genetic alterations, which limits the accuracy of these predictions (5, 6). Studies have shown significant differences in gene expression profiles and genetic alterations in DLBCL, leading to significant variation in clinical presentation and response to therapy (7). For instance, germinal center B-cell-like (GCB) and activated B-cell-like (ABC) subtypes, based on gene expression profiling, has been foundational in understanding the prognosis of DLBCL, and DLBCL patients with ABC subtype generally have worse outcomes compared to those with the GCB subtype (8, 9). In recent years, more genetic subtypes of DLBCL have been identified, providing deeper understanding and therapeutic directions (10, 11). Additionally, some prognostic models linked the immune-related genes and tumor microenvironment-related genes have also been developed, enabling more precise guidance for personalized treatment in DLBCL patients (12, 13).

Mitochondria-related genes (MitoRGs) play crucial roles in normal physiological processes and function including the regulation of cellular energy production, reactive oxygen species (ROS) management, calcium homeostasis, lipid biosynthesis and modulation of redox signaling pathways (14, 15). In tumor development, alterations in mitochondrial function and MitoRGs expression are recognized as important contributors to cancer progression (16). A key factor in this process is the Warburg effect (or aerobic glycolysis), where cancer cells primarily rely on glycolysis for energy production, even in the presence of oxygen. This metabolic reprogramming contributes to the proliferation, metastasis and chemoresistance of tumor cells, commonly observed in many types of cancer (17). In DLBCL, dysfunction of MitoRGs has become a key focus of research due to the important role mitochondria play in cellular metabolism, apoptosis, and inflammation. For instance, B-cell CLL/Lymphoma 2 (BCL2), a well-known MitoRG, is associated with double-hit, triple-hit and double expressor DLBCL, which are linked to a particularly aggressive clinical course and poor prognosis (18). Therefore, studying MitoRGs in DLBCL could provide valuable insights into the molecular mechanisms of the disease and open new pathways for targeted therapies focused on mitochondrial dynamics.

However, a prognostic signature based on MitoRGs for the survival of DLBCL patients has not yet been established or reported. To address this gap, we developed a novel prognostic signature based on MitoRGs for DLBCL patients, which offers new insights into the role of MitoRGs.

## Materials and methods

### Data source and preprocessing methods

**Figure 1:**
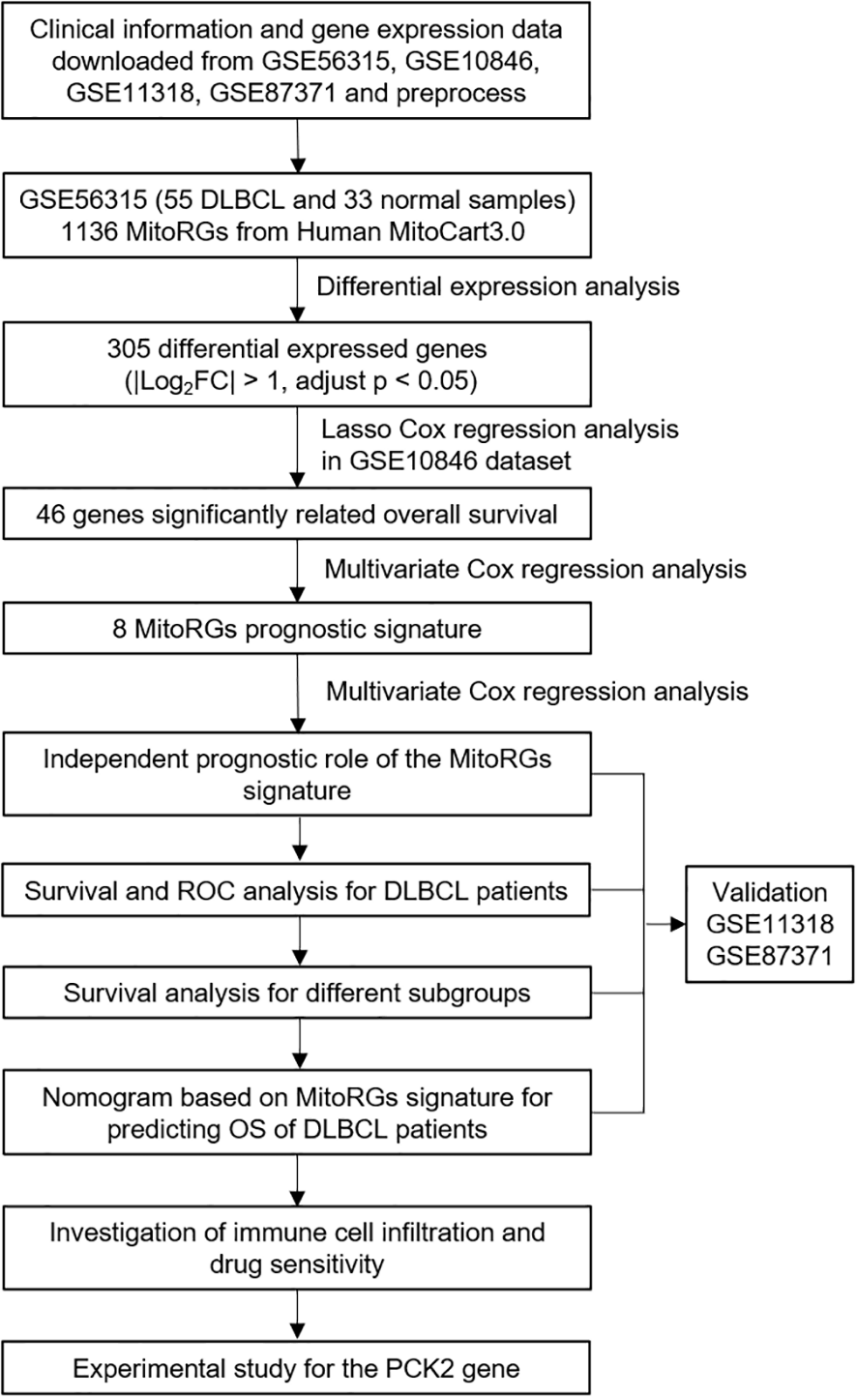
Flowchart of the study.

The workflow diagram of this study is shown in **Figure 1**. A total of 1136 mitochondria-related genes (MitoRGs) were obtained from the MitoCart3.0 database (https://www.broadinstitute.org/mitocarta) (19). Clinical data and gene expression profiles of DLBCL patients from four datasets (GSE56315, GSE10846, GSE11318 and GSE87371) were downloaded from the Gene Expression Omnibus (GEO) database (https://www.ncbi.nlm.nih.gov/geo/). 33 normal B-cell samples from the GSE56315 dataset include subtypes: naïve B-cells, centrocytes B-cells, centroblast B-cells, memory B-cells, and plasmablasts B-cells, all derived from healthy human tonsil tissue. Our analysis grouped these normal B-cell subtypes together as a single control group to provide a broad comparison against 55 DLBCL samples in GSE56315.

GSE10846 was served as the training cohort, whereas GSE11318 and GSE87371 datasets were designated as the validation cohorts. The “sva” R package was used to remove batch effects of the four GSE datasets.

### Analysis for the differentially expressed MitoRGs in DLBCL patients

Differential expression analysis of 1136 MitoRGs in the GSE56315 dataset was performed using “Limma” R package. The screening conditions were set as |Log_2_Fold Change (FC)| > 1 and adjust. p-value < 0.05. The “ComplexHeatmap” R package was used to visualize the differential genes, and the “ggplot” R package was used to draw the volcano map of these genes.

### Construction of the prognostic MitoRGs signature for the DLBCL patients

We conducted a Least Absolute Shrinkage and Selection Operator (Lasso) Cox regression analysis on the GSE10846 dataset to identify the MitoRGs associated with overall survival (OS) in DLBCL patients. Furthermore, we applied multivariate Cox regression analysis to identify prognostic genes and obtained the regression coefficient (coef) and hazard ratio (HR) for each gene. We calculated the prognostic risk score for each patient using the formula: risk score = Σ (expression of gene x coef). Using the “survminer” R package, we classified DLBCL patients into low-risk and high-risk groups based on the optimal cut-off values of the risk score. Kaplan-Meier survival curves were used to investigate the survival difference between the low-risk and the high-risk groups. The sensitivity and specificity of the prognostic model were examined by the Receiver Operating Characteristic (ROC) curve. Subsequently, the univariate and multivariate Cox regression analyses were used to evaluate the independent prognostic role of MitoRGs signature and multiple clinical factors, such as age, gender, DLBCL subtype, IPI score. The validation cohorts were used to confirm the previous analyses.

### Analyses for the immune infiltration, immune checkpoint expression and drug sensitivity

We performed single-sample gene set enrichment analysis (ssGSEA) using the ‘GSVA’ R package to compare immune infiltration in DLBCL patients across different risk groups. This analysis calculated the infiltration scores for 24 immune cells. Next, we analyzed the expression of immune checkpoints between the two groups. Additionally, we utilized the ‘pRRophetic’ R package, along with gene expression data from the Genomics of Drug Sensitivity in Cancer (GDSC) (https://www.cancerrxgene.org/) (20), to predict drug sensitivity in patients from both groups.

### Construction of the nomogram based on the prognostic MitoRGs signature

A nomogram was created using the risk score from the MitoRGs signature, age, and IPI score, based on the GSE10846 dataset with the “rms” R package. The sensitivity and specificity of the nomogram in predicting the 1-, 3-, and 5-year survival of DLBCL patients were analyzed using the ROC curve. Calibration curves and decision curves analysis (DCA) were employed to assess the reliability and clinical practicality of the model, respectively. The validation cohorts were utilized to confirm the prognostic significance of the nomogram.

### Study for the PCK2 gene

To explore the functional role of MitoRGs within the signature, univariate Cox regression analysis was conducted to assess the impact of individual genes on the OS of DLBCL patients in the GSE10846 dataset, and the PCK2 gene was investigated in the subsequent study. In addition, GEPIA 2 database (http://gepia2.cancer-pku.cn/) (20) was used to compare the expression of PCK2 gene in 47 DLBCL patients and 337 normal samples. The Human Protein Atlas (HPA) database (https://www.proteinatlas.org/) (21) was used to investigate the expression of PCK2 gene in multiple cancer cell lines.

### Cells culture

DLBCL cell lines (U-2932, SU-DHL4, SU-DHL6) were sourced from the department of hematology, West China Hospital, Sichuan University, and were purchased from ATCC by professor Yongqian Jia. DLBCL cells were cultured in the RPMI-1640 (BasalMedia Technologies, China, L210KJ) medium, and human embryonic kidney 293T (293-T) cells were cultured in the DMEM (BasalMedia Technologies, China, L110KJ) medium, supplemented with 10% fetal bovine serum (FBS) and antibiotics (100 U/mL penicillin and 0.1 mg/mL streptomycin) at 37°C in a humidified incubator containing 5% CO_2_. For high- and low-glucose experiments, glucose-free RPMI-1640 with 2mM glutamine (BasalMedia Technologies, China, L270KJ) was supplemented with 20/1 mM (high/low) glucose (BasalMedia Technologies, China, S261JV), 10% FBS and antibiotics.

### PCK2 knockdown and real-time quantitative polymerase chain reaction assay

Small interfering RNAs (siRNAs) targeting PCK2, as well as a negative control (si-NC), were synthesized and purchased from Tsingke (Beijing, China). The transfection reagent EZ trans siRNA (Life-iLab, Shanghai, China, AC04L051) was utilized in accordance with the manufacturer’s instructions to knock down PCK2.

Total RNA was extracted using the RNA extraction kit (Tiangen, Beijing, China, DP451), and reverse transcribed into cDNA using the FastKing RT Kit (Tiangen, Beijing, China, KR116). The expression of PCK2 was quantitatively detected by the RT-qPCR assay using the SYBR Green Pro Taq HS Mix (Accurate Biology, Changsha, Hunan AG11701). Primers for PCK2 as follows: forward primer: TGCCAGGCTGGAAAGTGGAGTGT; reverse primer: GCAACCCCAAAGAAGCCGTTCTCA. GAPDH was used as an inner control with forward primer: AATGAAGGGGTCATTGATGG; reverse primer: AAGGTGAAGGTCGGAGTCAA. The relative expression of PCK2 was calculated using the 2^-ΔΔct^ method.

### Cell counting kit-8 assay and cell apoptosis analysis

Following 48 hours of PCK2 knockdown or not, DLBCL cells were incubated under different culture conditions for 24, 48, and 72 hours, 10 μl of CCK-8 reagent (Oriscience, Chengdu, China, CB101) was added to each well and incubated for an additional three hours. The optical density (OD) value was measured at 450 nm absorbance to detect the cell proliferation using the Tecan Infinite M Nano reader (Tecan Group Ltd, Männedorf, Switzerland).

To detect the cell apoptosis, DLBCL cells, with or without PCK2 knockdown, were incubated for another 48 hours in distinct culture conditions. Cells were collected and washed once with cold PBS, and stained with Annexin V and PI solution (Abbkine, Wuhan, China, KTA0002) at room temperature for 15 minutes, avoiding light exposure. The percentage of apoptotic cells was assessed using the ACEA NovoCyte flow cytometer (Agilent Technologies, Santa Clara, California, USA).

### Statistical analyses

The R software (v4.4.0) was utilized for statistical analyses and plotting. Univariate and multivariate Cox regression analyses were performed to evaluate the prognostic value of clinical factors and risk model for DLBCL patients. Chi-Square test was used to investigate the relationship between different risk groups and clinical factors. Comparisons of numeric data between or among groups were analyzed using the student’s t-test, ANOVA test or Mann-Whitney U test, whichever was available. p < 0.05 was the threshold for significance.

## Results

### Differentially expressed MitoRGs in DLBCL patients

**Figure 2:**
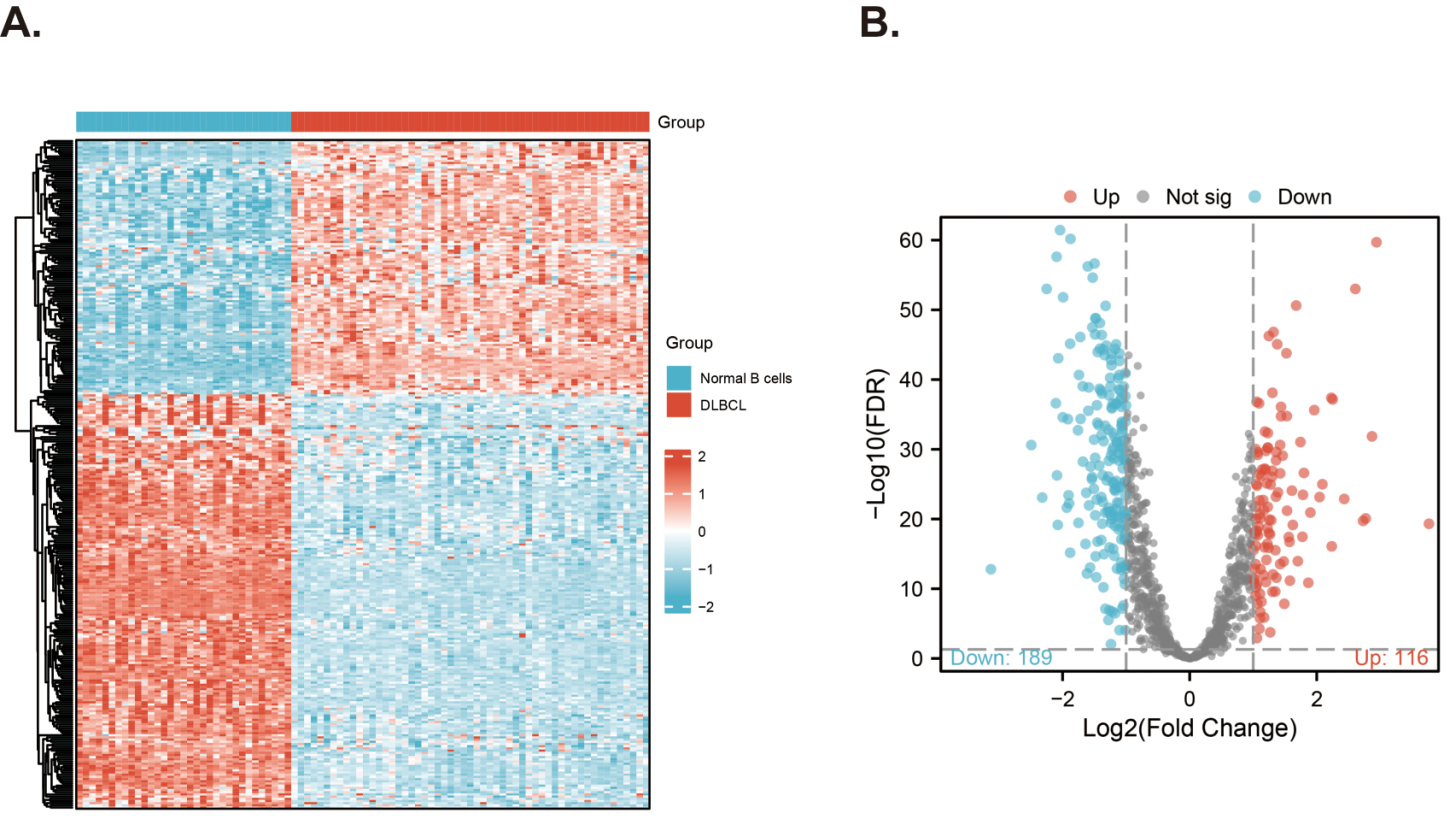
Screening for differentially expressed MitoRGs in the GSE56315 dataset. **(A)** The heatmap shows the differentially expressed mitochondrial-related genes. **(B)** The Volcano plots of the differentially expressed mitochondrial-related genes (MitoRGs). FDR, False Discovery Rates.

The clinical information of the samples from the GSE datasets used in this study is displayed in **Table 1**. We downloaded 1136 MitoRGs from the MitoCart3.0 database to identify differentially expressed MitoRGs in DLBCL patients. After matching these genes with the GSE56315 dataset, we identified a total of 1096 MitoRGs. We compared the expression levels of the 1096 MitoRGs in 55 DLBCL samples and 33 normal B cell samples from GSE56315. This analysis revealed 305 differentially expressed genes, with 116 up-regulated and 189 down-regulated, as shown in the heatmap (**Figure 2A**) and volcano plot (**Figure 2B**).

**Table 1:** Clinical information of DLBCL patients from the GSE datasets.

	GSE10846 (n=414)	GSE11318 (n=200)	GSE87371 (n=221)
Age (y), (Median, IQR)	62.5 (14-92)	64 (14-88)	60 (19-87)
Gender			
Female/Male/NA	172/224/18	110/90	116/105
Subtype			
GCB/ABC/PMBL/Unclassified	183/167/NA/64	70/73/30/27	84/83/20/34
Stage			
I//II/III/IV/NA	66/122/97/121/8	25/50/32/55/38	
LDH ratio			NA
Normal/Elevated/NA	173/178/63	68/76/56	
ECOG			NA
<2 /≥2/NA	296/93/25	122/39/39	
Extra-nodal sites			NA
≤1/ >1/NA	353/30/31	134/28/38	
IPI			
0-1/2/3/4-5/NA	132/96/59/34/93	54/39/28/21/58	74/45/53/46
Treatment regimen			
CHOP-like	181	220	0
R-CHOP-like	233	0	179
ACVBP-like	0	0	6
R-ACVBP -like	0	0	36
Status			
Dead/Alive	165/249	112/88	53/168

NA, Not available; IQR, Interquartile Range; GCB, Germinal Center B-cell-like DLBCL; ABC, Activated B-cell-like DLBCL; PMBL, Primary Mediastinal B-cell Lymphoma; CHOP, Cyclophosphamide + Doxorubicin +Vincristine + Prednisone; R-CHOP, Rituximab + CHOP regimen; ACVBP, Doxorubicin +Cyclophosphamide + Vincristine + Bleomycin + Prednisone; R-ACVBP, Rituximab + ACVBP regimen.

### Construction of the prognostic MitoRGs signature

**Figure 3:**
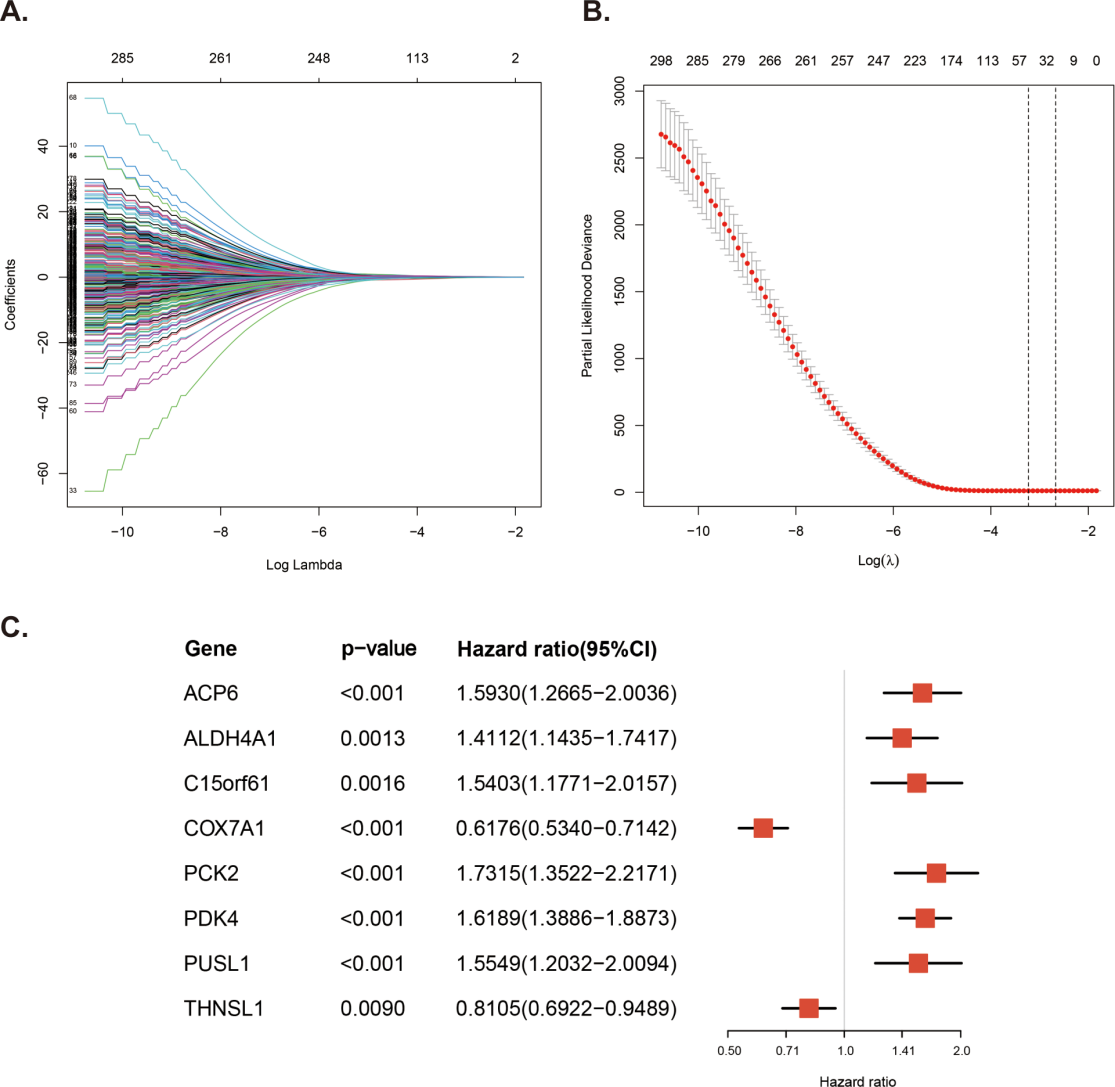
Construction of the prognostic MitoRGs signature. **(A)** Lasso coefficient profiles plots coefficient paths of 305 candidate MitoRGs associated OS of DLBCL patients. **(B)** Partial likelihood deviance from cross-validation plotted against log lambda values, with dashed lines representing the optimal lambda values. **(C)** Forest plots of the multivariate Cox regression analyses for the eight MitoRG significantly associated OS of DLBCL patients.

To screen the MitoRGs related to the overall survival (OS) of DLBCL patients, we used Lasso Cox regression analysis to obtain 46 MitoRGs (**Figures 3A, B**). Further, we used multivariate Cox regression analysis to obtain eight genes suitable for the MitoRGs signature (**Figure 3C**). In multivariate Cox regression, all the variables satisfied the proportional hazards (PH) hypothesis (p > 0.05) and multicollinearity of covariates (variance inflation factor < 2). We calculated each patient’s risk score using the expression levels of the eight identified genes and their corresponding coef from the multivariate Cox regression analysis. The formula is as follows: Risk score = (ACP6 x 0.4656) + (ALDH4A1 x 0.34447) + (C15orf61 x 0.432) – (COX7A1 x 0.48195) + (PCK2 x 0.54897) + (PDK4 x 0.48174) + (PUSL1 x 0.44141) - (THNSL1 x 0.21016).

### Predictive role of MitoRGs signature for OS of DLBCL patients

**Figure 4:**
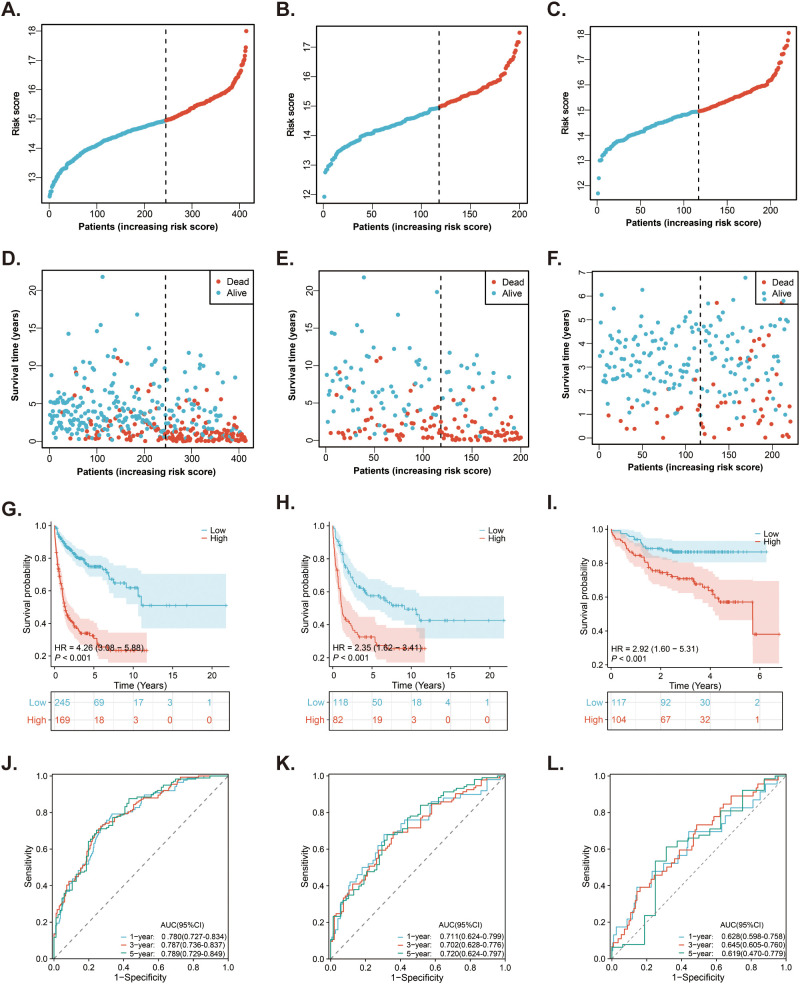
Prediction of the prognostic MitoRGs signature in DLBCL patients. **(A–C)** The distribution of risk scores in the training cohort (GSE10846) and validation cohorts (GSE11318 and GSE87371). **(D–F)** The distribution of survival time and status of patients in the training and validation cohorts. **(G–I)** Kaplan-Meier survival curves comparing OS between low- and high-risk groups in the training and validation cohorts. **(J–L)** Time-dependent ROC curves for predicting 1-, 3-, and 5-year OS in the training cohort and validation cohorts, with corresponding AUC values and 95% confidence intervals (95%CI).

Based on the risk score and optimal cut-off value derived from the MitoRGs signature, we categorized the DLBCL patients in the GSE10846 dataset into low-risk (n=245) and high-risk (n=169). The distribution of risk score for patients is depicted in **Figure 4A**. Additionally, there were more death events as the risk score increased (**Figure 4D**). Kaplan-Meier survival analysis revealed that the survival of patients in the high-risk group was significantly shorter than that in the low-risk group (HR=4.26, p < 0.001) (**Figure 4G**). The above results were verified in the GSE11318 dataset (**Figures 4B, E, H**) and GSE87371 dataset (**Figures 4C, F, I**). In order to test the predictive effect of MitoRGs signature on OS of patients, ROC curve analysis was performed. In GSE10846 dataset, the area Under the Curve (AUC) of MitoRGs signature for predicting 1-, 3-, and 5- years OS was as follows: 0.780 (95%CI 0.727-0.784), 0.787 (95%CI 0.736-0.837) and 0.789 (95%CI 0.729-0.849) (**Figure 4J**). The validation analysis in the validation dataset also suggested good prediction results (**Figures 4K, L**).

### Independent prognostic role of MitoRGs signature for OS of DLBCL patients

**Figure 5:**
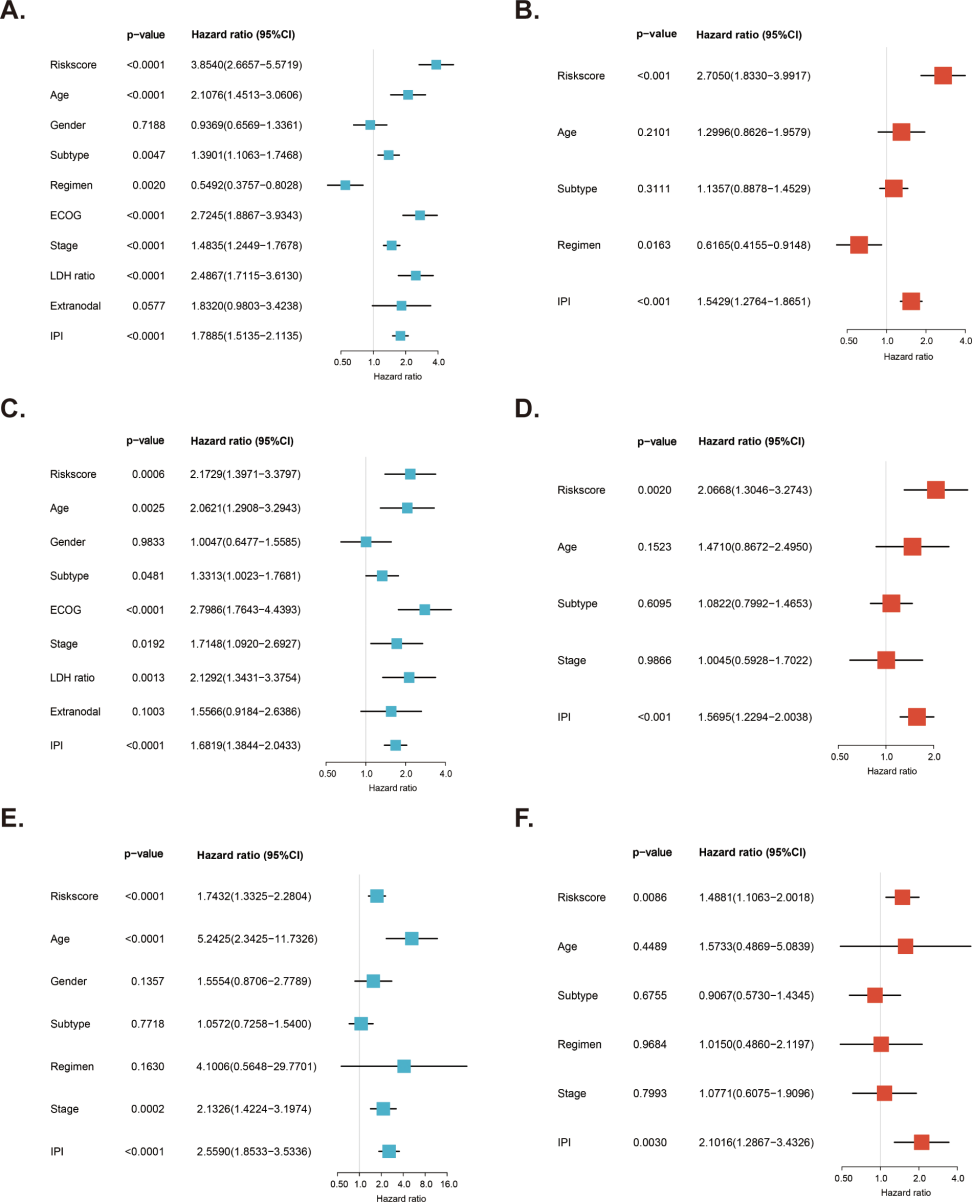
Independent prognostic role of MitoRGs signature for OS of DLBCL patients. **(A, C, E)** Forest plots showing the univariate Cox regression analyses of clinical parameters and risk score in training cohort (GSE10846) and validation cohorts (GSE11318 and GSE87371). **(B, D, F)** Forest plots of the multivariate Cox regression analyses of clinical parameters and risk score in training cohort and validation cohorts.

We examined the independent prognostic role of the MitoRGs signature in the OS of DLBCL patients by cleaning the clinical data from the training cohort and two validation cohorts, excluding those with incomplete information and primary mediastinal B-cell lymphoma (PMBL) subtype due to its unique molecular biological features. Next, we analyzed the relationship between clinical information of patients and the risk score (**Table 2**). In the GSE10846 dataset, a higher proportion of low-risk patients were aged ≤ 60 years, had the GCB subtype, were at stage I/II, exhibited a normal lactate dehydrogenase (LDH) ratio, had ≤ 1 extra-nodal site, scored 0-2 on the IPI, and were alive. For the GSE11318 dataset, the proportion of patients in the low-risk group with GCB subtype and being alive was also higher than that in the high-risk group. Additionally, patients with age ≤ 60 years, GCB subtype, stage I/II, IPI 0-2 score, and being alive were more frequently observed in the low-risk group in the GSE87371 dataset. Subsequently, we analyzed the risk score alongside multiple clinical factors in both the training cohort and validation cohorts. Univariate Cox regression was used to analyze the influence of various factors on the OS of DLBCL patients, and it was found that the risk score was one of the important factors (**Figures 5A, C, E**). Furthermore, we selected four factors, including the risk score, age, subtype, and IPI score, for multivariate Cox regression analysis in these three datasets. The findings revealed that the risk score of the MitorRGs signature served as an independent prognostic factor for OS in DLBCL patients, with HRs of 2.7050, 2.0668, and 1.4881 (all p<0.05) (**Figures 5B, D, F**).

**Table 2:** Association between clinical parameters of DLBCL patients and MitoRGs signature.

	GSE10846 (n=306)			GSE11318 (n=142)			GSE87371 (n=201)		
	Low risk (n=183)	High risk (n=123)	p-value	Low risk (n=82)	High risk (n=60)	p-value	Low risk (n=100)	High risk (n=101)	p-value
Age, n (%)			**0.006**			0.486			**0.002**
≤ 60y	99 (54.1%)	47 (38.2%)		39 (47.6%)	25 (41.7%)		58 (28.9%)	37 (18.4%)	
> 60y	84 (45.9%)	76 (61.8%)		43 (52.4%)	35 (58.3%)		42 (20.9%)	64 (31.8%)	
Gender, n (%)			0.615			0.969			0.229
Female	78 (42.6%)	56 (45.5%)		38 (46.3%)	28 (46.7%)		44 (21.9%)	53 (26.4%)	
Male	105 (57.4%)	67 (54.5%)		44 (53.7%)	32 (53.3%)		56 (27.9%)	48 (23.9%)	
Subtype, n (%)			**< 0.001**			**0.022**			**< 0.001**
GCB	96 (31.4%)	38 (12.4%)		42 (29.6%)	18 (12.7%)		55 (27.4%)	29 (14.4%)	
ABC	59 (19.3%)	66 (21.6%)		26 (18.3%)	32 (22.5%)		25 (12.4%)	58 (28.9%)	
Unclassified	28 (9.2%)	19 (6.2%)		14 (9.9%)	10 (7%)		20 (10%)	14 (7%)	
Stage, n (%)			**0.006**			0.260			**0.004**
I-II	98 (53.6%)	46 (37.4%)		42 (51.2%)	25 (41.7%)		38 (18.9%)	20 (10%)	
III-IV	85 (46.4%)	77 (62.6%)		40 (48.8%)	35 (58.3%)		62 (30.8%)	81 (40.3%)	
LDH ratio, n (%)			**< 0.001**			0.071	NA	NA	
Normal	109 (59.6%)	48 (39%)		44 (53.7%)	23 (38.3%)				
Elevated	74 (40.4%)	75 (61%)		38 (46.3%)	37 (61.7%)				
ECOG, n (%)			**< 0.001**			0.515	NA	NA	
< 2	151 (82.5%)	80 (65%)		64 (78%)	44 (73.3%)				
> 2	32 (17.5%)	43 (35%)		18 (22%)	16 (26.7%)				
Extra-nodal, n (%)			0.223			0.802	NA	NA	
≤ 1	172 (94%)	111 (90.2%)		67 (81.7%)	50 (83.3%)				
> 1	11 (6%)	12 (9.8%)		15 (18.3%)	10 (16.7%)				
IPI, n (%)			**< 0.001**			0.345			
0-1	93 (50.8%)	33 (26.8%)		36 (43.9%)	18 (30%)		41 (20.4%)	21 (10.4%)	**< 0.001**
2	52 (28.4%)	38 (30.9%)		20 (24.4%)	19 (31.7%)		25 (12.4%)	16 (8%)	
3	29 (15.8%)	28 (22.8%)		16 (19.5%)	12 (20%)		20 (10%)	29 (14.4%)	
4-5	9 (4.9%)	24 (19.5%)		10 (12.2%)	11 (18.3%)		14 (7%)	35 (17.4%)	
Treatment, n (%)			**0.003**	NA	NA				**0.043**
CHOP-like	72 (39.3%)	70 (56.9%)					NA	NA	
R-CHOP-like	111 (60.7%)	53 (43.1%)					49 (24.4%)	67 (33.3%)	
ACVBP-like	NA	NA					9 (4.5%)	5 (2.5%)	
R-ACVBP-like	NA	NA					42 (20.9%)	29 (14.4%)	
Status, n (%)			**< 0.001**			**0.013**			**< 0.001**
Dead	46 (25.1%)	77 (62.6%)		39 (47.6%)	41 (68.3%)		12 (6%)	36 (17.9%)	
Alive	137 (74.9%)	46 (37.4%)		43 (52.4%)	19 (31.7%)		88 (43.8%)	65 (32.3%)	

NA, Not available; GCB, Germinal Center B-cell-like DLBCL; ABC, Activated B-cell-like DLBCL; CHOP, Cyclophosphamide + Doxorubicin +Vincristine + Prednisone; R-CHOP, Rituximab + CHOP regimen; ACVBP, Doxorubicin +Cyclophosphamide + Vincristine + Bleomycin + Prednisone; R-ACVBP, Rituximab + ACVBP regimen.

Bold values mean statistically significant difference.

### Prognostic performance of the MitoRGs signature in different subgroups of DLBCL patients

**Figure 6:**
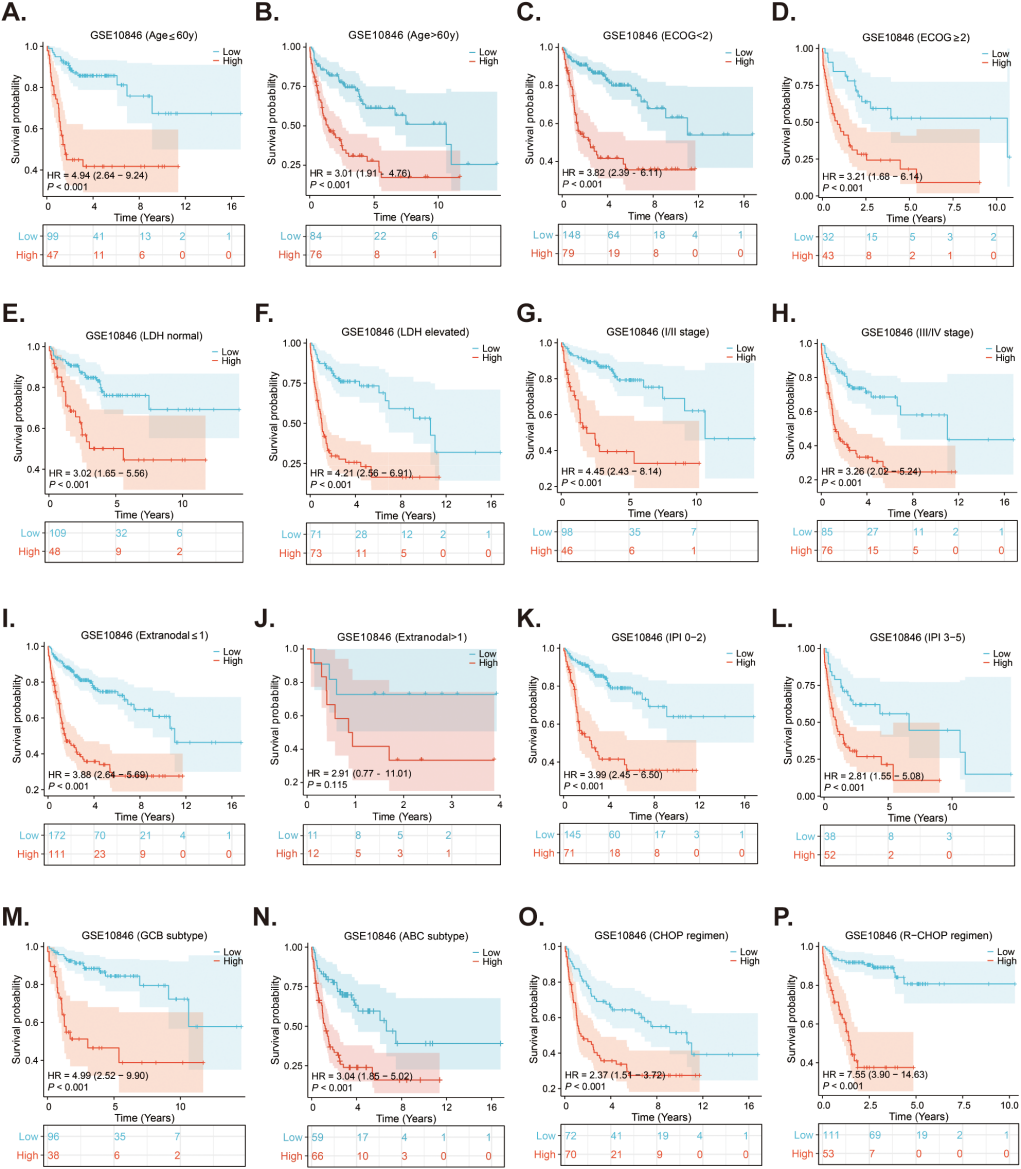
Subgroups survival analyses of the prognostic MitoRGs signature in GSE10846. **(A, B)** The Kaplan-Meier curves of low- and high-risk score in patients younger and older than 60-year-old. **(C, D)** The Kaplan-Meier curves of low- and high-risk score in ECOG < 2 scores and ≥2 scores subgroup. **(E, F)** The Kaplan-Meier curves of low- and high-risk score in LDH normal and LDH elevated subgroup. **(G, H)** The Kaplan-Meier curves of low- and high-risk score in I/II stage and III/IV stage subgroup. **(I, J)** The Kaplan-Meier curves of low- and high-risk score in extra-nodal sites ≤1 and >1 subgroup. **(K, L)** The Kaplan-Meier curves of low- and high-risk score in IPI 0-2 scores and IPI 3-5 scores subgroup. **(M, N)** The Kaplan-Meier curves of low- and high-risk score in GCB and ABC subgroup. **(O, P)** The Kaplan-Meier curves of low-risk score and high-risk score in CHOP and R-CHOP regimen treated patients.

As the survival of DLBCL patients is affected by many factors, such as age. Therefore, Kaplan-Meier survival analyses were conducted in the GSE10846 dataset to evaluate the predictive ability of the risk score for OS in different subgroups of DLBCL patients. As presented in **Figure 6**, except for the subgroup of extra-nodal sites > 1, the OS of patients in the high-risk group was significantly shorter than that of the low-risk group across various subgroups, including age, ECOG score, LDH ratio, stage, IPI score, subtype, and treatment regimen (all p < 0.05). Additionally, subgroup survival analyses were conducted using the validation cohorts GSE11318 (**Supplementary Figure S1**) and GSE87371 (**Supplementary Figure S2**), which confirmed that the risk score had strong predictive capability for OS in various subgroups of DLBCL patients.

### MitoRGs signature supplements the prognostic role of IPI score in DLBCL

**Figure 7:**
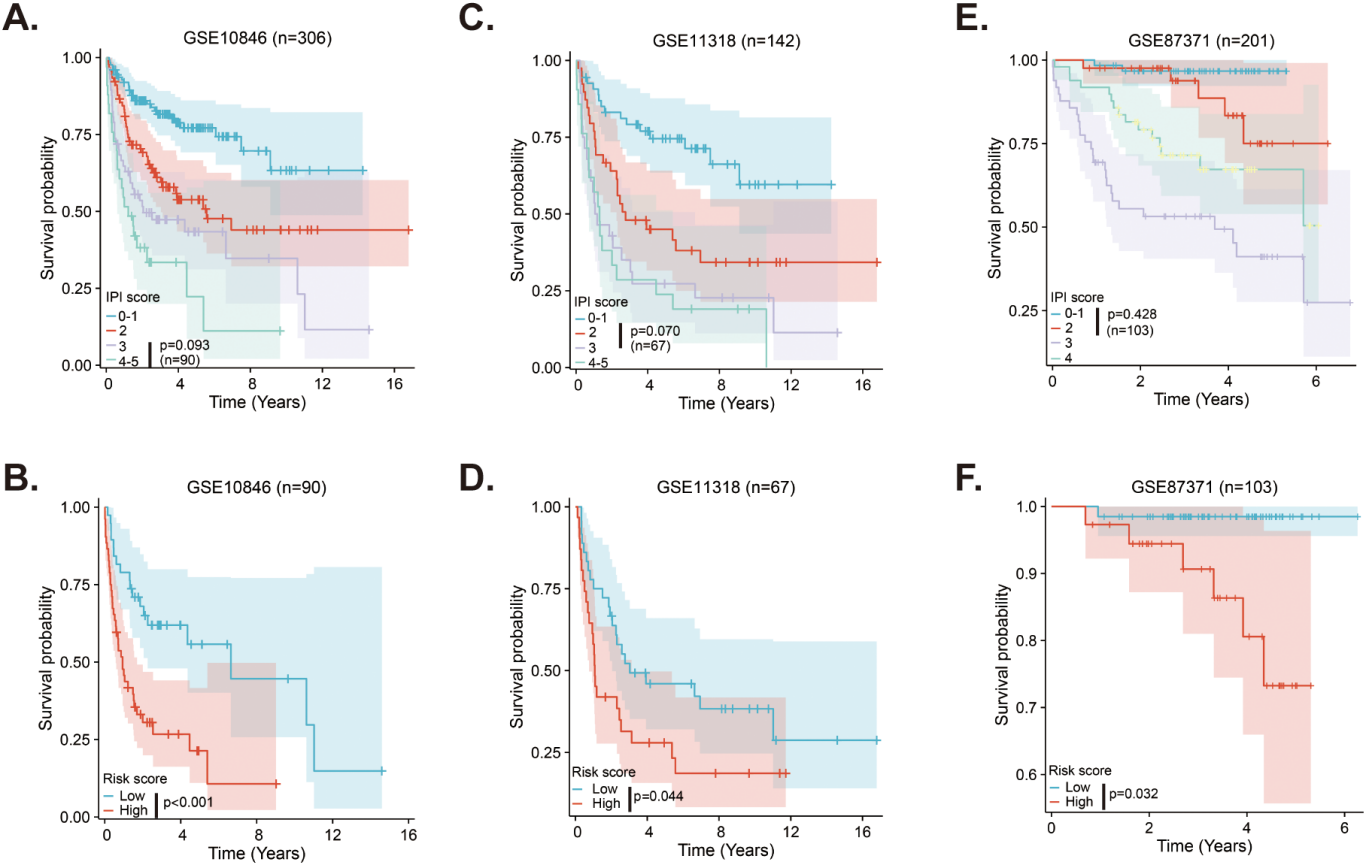
MitoRGs signature acts as the supplement to the IPI score for the prognostic role in DLBCL patients **(A–C)**. The Kaplan-Meier curves of DLBCL patients with different IPI score in training cohort (GSE10846) and validation cohorts (GSE111318 and GSE87371). **(D–F)** The Kaplan-Meier curves based on the risk score of the MitoRGs signature predicted the prognosis accurately for patients with less distinction by IPI score in training cohort (GSE10846) and validation cohorts (GSE111318 and GSE87371).

The IPI score is the most commonly used method for evaluating the prognosis of DLBCL patients in clinical settings, but it does not always accurately distinguish between all patient. In the GSE10846 dataset, Kaplan-Meier curves using the IPI score could not differentiate survival between patients with IPI scores of 3 and those with scores of 4-5, whereas the risk score of the MitoRGs signature was able to do so (**Figures 7A, D**). Similar results were found in the validation cohorts. The Kaplan-Meier curves of the IPI scores with 2 and 3 in the GSE11318, and the IPI scores with 0-1 and 2 in the GSE87371 could not predict the prognosis of these patients (**Figures 7B, C**). However, the risk score of the MitoRGs signature could also exactly assess their survival (**Figures 7E, F**). These findings suggest that the MitoRGs signature could supplement the prognostic role of the IPI score in DLBCL patients.

### Investigation of immune cell infiltration and drug sensitivity

**Figure 8:**
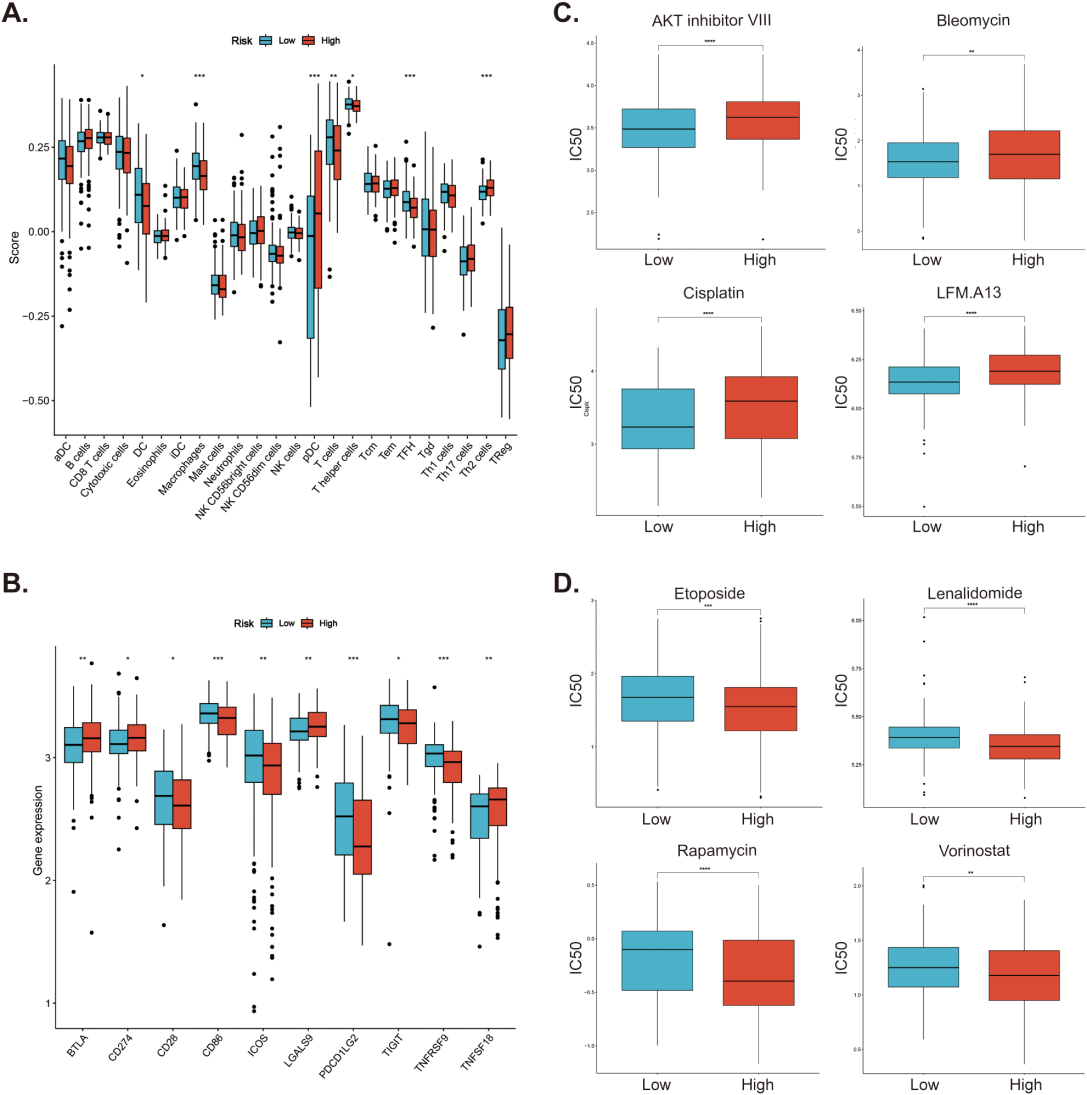
Investigation of immune cell infiltration and drug sensitivity in low- and high-risk patients in GSE10846 dataset. **(A)** Immune cell infiltration in the low- and high-risk patients. **(B)** Differential expression of immune checkpoints between low- and high-risk patients. **(C)** Low-risk patients. are more sensitive to these drugs **(D)** High-risk patients responded better to these drugs. *p < 0.05; **p < 0.01; ***p < 0.005; ****p < 0.001.

To investigate the immune cell infiltration in DLBCL patients in different risk groups, we used the ssGSEA method to analyze the infiltration of 24 immune cells in different groups of patients. The results suggested that patients in the high-risk group had less infiltration of macrophages, dendritic cells (DCs), T helper cells, T follicular helper (TFH) cells, while had more plasmacytoid dendritic cells (pDCs) and Th2 cells (**Figure 8A**). Then, for the expression of immune checkpoints, the expression of CD28, CD86, ICOS, PDCD1LG2 (PD-L2), TIGIT, and TNFRSF9 was higher in the low-risk group, and BTLA, CD274 (PD-L1), LGALS9, and TNFSF18 was higher in the high-risk group (**Figure 8B**). Subsequently, we further analyzed the sensitivity of patients in the low- and high-risk groups to various potential therapeutic agents and presented several representative therapeutic agents. As shown in **Figure 8C**, patients in the low-risk group responded better to AKT inhibitor VIII, Bleomycin, Cisplatin, and LFM.A13, while those in the high-risk group were more sensitive to Etoposide, Lenalidomide, Rapamycin, and Vorinostat (**Figure 8D**).

### Establishing of the nomogram

**Figure 9:**
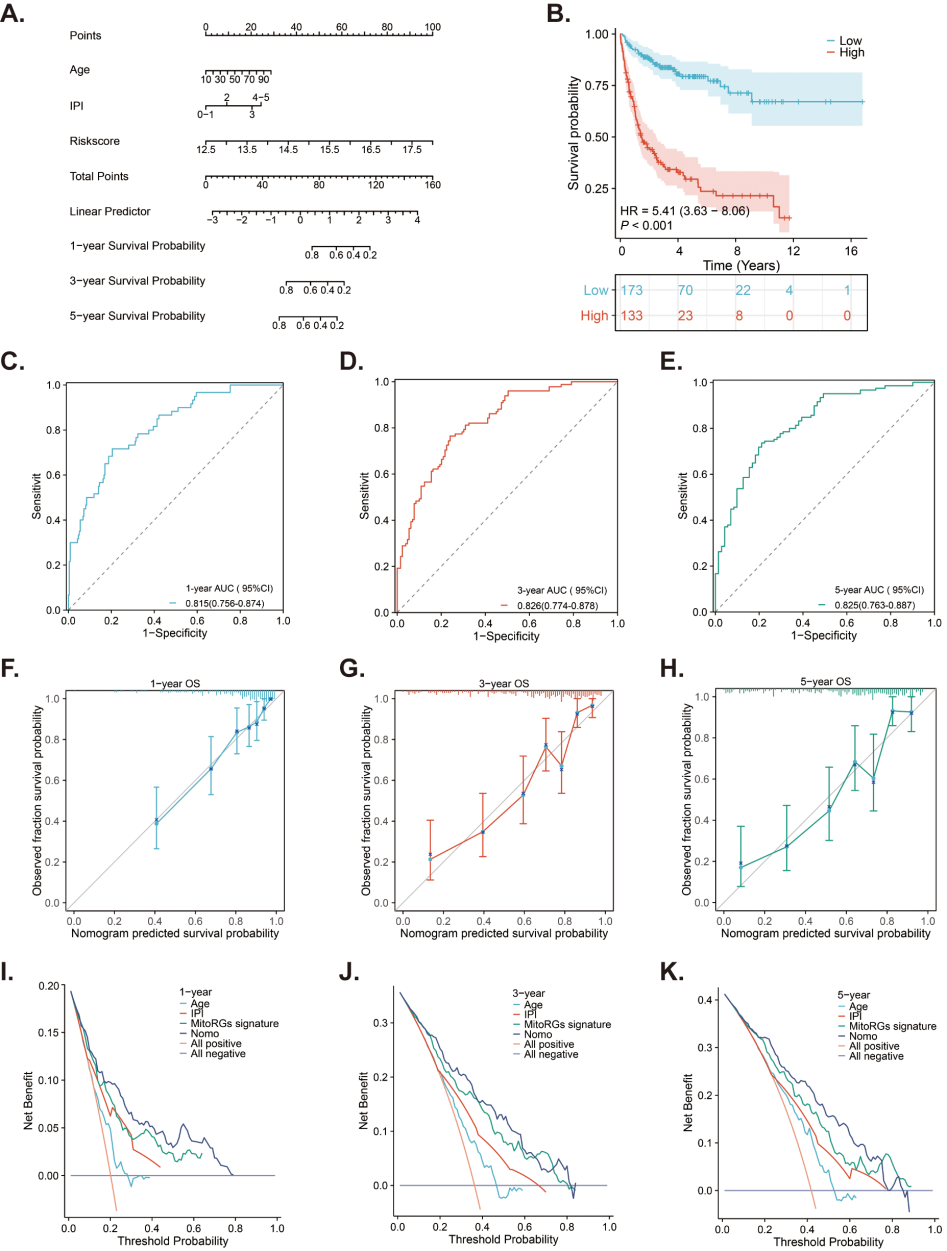
A nomogram developed from the MitoRGs signature to predict the OS of DLBCL patients. **(A)** A nomogram model predicted the 1-, 3- and 5-year OS in the training cohort (GSE10846). **(B)** Kaplan-Meier curves displaying patients with low- and high-risk scores derived from the nomogram model. **(C–E)** Time-dependent ROC curves of the nomogram model for predicting 1-, 3-, and 5-year OS, along with the corresponding AUC values and 95%CI. **(F–H)** The calibration curves of the nomogram model predicting 1-, 3-, and 5-year OS. **(I–K)** The Decision curve analysis (DCA) curves of age, IPI score, MitoRGs signature and the nomogram model predicting 1-, 3-, and 5-year OS.

A nomogram is a tool that predicts patient prognosis by combining various clinical factors. We selected age, IPI score, and risk score from the MitoRGs signature in the GSE10846 dataset. These factors were used to construct a nomogram to predict the 1-, 3-, and 5-year prognosis of DLBCL patients. The results indicated that risk score significantly contributes to the total score of the nomogram (**Figure 9A**). Using the new risk score from the nomogram, we can categorize DLBCL patients into low-risk and high- risk groups. Patients in the high-risk group had a significantly shorter OS compared to those in the low-risk group (HR = 5.41, p < 0.001) (**Figure 9B**). ROC curve analyses indicated that the nomogram effectively predicts the 1-, 3-, and 5-year OS of DLBCL patients, with AUC values of 0.815 (95%CI 0.756-0.874), 0.826 (95%CI 0.774-0.878), and 0.825 (95%CI 0.763-0.887), respectively (**Figures 9C–E**). The calibration curve analyses further confirmed the predictive accuracy of the nomogram (**Figures 9F–H**). DCA decision analyses were used to evaluate the clinical utility of the nomogram, and the results suggested that it outperformed age, IPI score, and MitoRGs signature alone (**Figures 9I–K**). These findings were further confirmed in the validation cohorts, yielding similar results (**Supplementary Figure S3**).

### Study for the PCK2 gene

**Figure 10:**
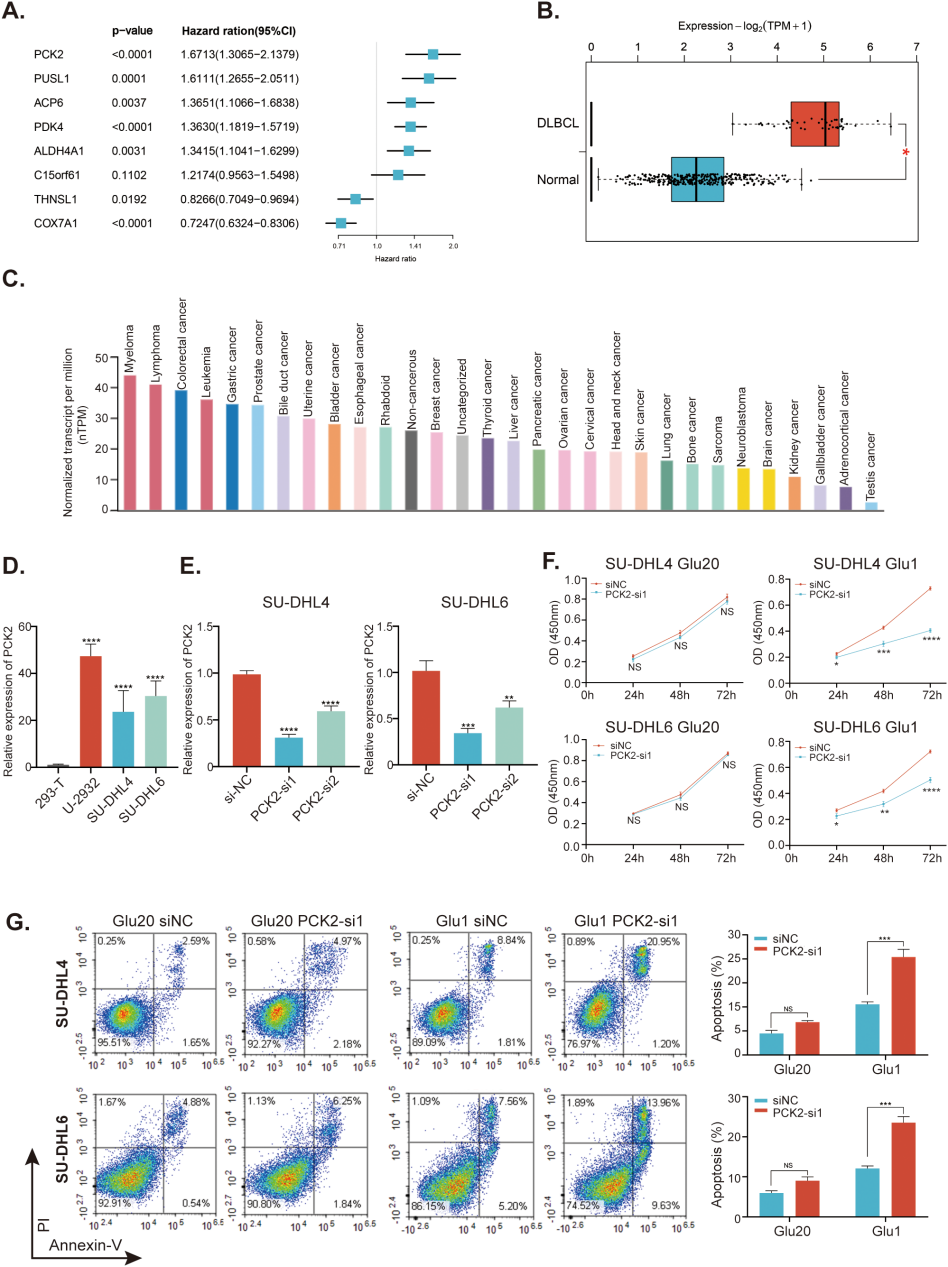
Study for the mitochondrial-related gene PCK2. **(A)** Forest plots of the univariate Cox regression analyses of the eight genes within the MitoRGs signature for the OS of DLBCL patients. **(B)** Expression of PCK2 between DLBCL patients and healthy individuals from the GEPIA2 database. **(C)** Expression of PCK2 in distinct cancer cell lines in the HPA database. **(D)** Expression of PCK2 in various DLBCL cell lines and 293T cells detected by RT-qPCR assay. **(E)** knockdown efficacy for PCK2 gene in SU-DHL4 and SU-DHL6 cell lines confirmed by RT-qPCR assay. **(F)** CCK-8 assay to investigate the proliferation of DLBCL cell lines with or without PCK2 knockdown in high- and low-glucose condition. **(G)** Flow cytometry to examine the apoptosis of DLBCL cell lines with or without PCK2 knockdown in high- and low-glucose condition. Glu20 refers to a culture condition with 20 mM glucose, while Glu1 indicates a 1 mM glucose condition. NS, No significance; *p < 0.05, **p < 0.01, ***p < 0.005, ****p < 0.001.

The result of univariate Cox regression analysis indicated that the PCK2 gene exhibited the highest HR (**Figure 10A**). Additionally, the GEPIA 2 database showed that PCK2 was significantly upregulated in DLBCL patients compared to heathy controls (**Figure 10B**). We then investigated the expression of PCK2 among multiple cancer cell lines and discovered significantly higher levels in lymphoma cell lines (**Figure 10C**). In comparison to 293T cells, PCK2 expression was significantly higher in three DLBCL cell lines: U-2932, SU-DHL4, SU-DHL6 (**Figure 10D**). We transfected SU-DHL4 and SU-DHL6 cells with specific siRNAs targeting PCK2, and observed that siRNA1 (PCK2-si1) exhibited superior silencing efficacy (**Figure 10E**). Afterward, we cultured SU-DHL4 and SU-DHL6 cells in a 20mM glucose environment, where PCK2 silencing did not affect the proliferation of cells. Conversely, in a 1mM glucose condition, PCK2 knockdown significantly inhibited proliferation in a time-dependent manner (**Figure 10F**). Moreover, cells apoptosis was assessed and the result revealed that PCK2 gene knockdown did not influence cell apoptosis under the high-glucose conditions; however, cells exhibited evident apoptosis in low- glucose conditions (**Figure 10G**).

## Discussion

Currently, several prognostic MitoRGs signatures have been developed in various cancer types (21–25). In this study, we developed a novel MitoRGs model to predict the prognosis of DLBCL patients. We first acquired differentially expressed MitoRGs by comparing DLBCL cells and normal B cells. Lasso Cox regression and multivariate Cox regression were performed to construct an eight-MitoRGs signature for predicting the OS of DLBCL patients. The signature has demonstrated robust performance and accuracy in predicting 1-, 3-, and 5-year OS of DLBCL patients across both training cohort and validation cohorts. Meanwhile, subgroup survival analyses based on several clinical parameters further supported the prognostic role of the MitoRGs signature. Importantly, the signature could supplement the prognostic role of IPI score in DLBCL patients. Additionally, we established a nomogram model that combines age, IPI score and the risk score from the signature to predict the prognosis of DLBCL patients. The nomogram model provided a better prognostic value and the results of DCA demonstrated its practicability in clinical settings.

The introduction of immunotherapy has greatly improved the prognosis for DLBCL patients, making the R-CHOP the standard first-line treatment (1). Immune cell infiltration plays a crucial role in the therapeutic response in DLBCL. Different types of immune cells contribute to the tumor microenvironment and influence treatment outcomes (26). According to the MitoRGs signature, DLBCL patients in the high-risk group exhibited less macrophages, DCs, T cells, T helper cells and TFH cells, while exhibiting an increase in pDCs and Th2 cells in the tumor microenvironment (TME). This altered immune landscape may contribute to the poor prognosis observed in these patients. A study found that high counts of CD68(+) macrophage were associated with improved progression-free survival (PFS) and OS for patients undergoing dose-dense chemoimmunotherapy, suggesting that macrophages may play a protective role in the TME of DLBCL (27). Conversely, another study indicated that low T-cell proportions in the TME, which often accompany reduced macrophage infiltration, are associated with immune escape and poor survival outcomes in DLBCL patients (28). Research indicates that the presence of TFH cells is crucial for effective humoral immune responses, as they play a significant role in regulating B cell activity and antibody production (29). In contrast, the reduction of TFH cells in high-risk DLBCL patients suggests a compromised ability to mount an adequate immune response against the tumor. Additionally, the skewing towards Th2 cells may indicate a shift in the immune response that favors humoral immunity over cellular immunity, potentially leading to tumor progression (30). Furthermore, the role of DCs in shaping T cell responses is well-documented. DCs can influence the differentiation of T cells into various subsets, including TFH cells, which are essential for germinal center formation and B cell activation (31). The decrease in DCs in high-risk DLBCL patients may impair the activation and differentiation of TFH cells, further exacerbating the immune evasion by the tumor (32). Moreover, the presence of pDCs has been associated with immune suppression in various cancers, including DLBCL. These cells can produce type I interferons and other cytokines that may promote regulatory T cell (Treg) expansion, thereby dampening anti-tumor immunity (33). The increased levels of pDCs in high-risk DLBCL patients could contribute to an immunosuppressive environment, facilitating tumor growth and survival (34). In summary, these results highlight a significant shift towards an immunosuppressive TME that may hinder effective anti-tumor responses in the DLBCL patients within the high-risk group. Understanding these dynamics is crucial for developing targeted therapies that can restore immune balance and improve patient outcomes. Furthermore, we also analyzed the expression of ICs in the DLBCL patients across various risk groups. The differentially expressed ICs suggested distinct immune evasion mechanisms and potential therapeutic strategies for each risk group. For instance, PD-L1 and PD-L2 are the ligands for PD-1, which suppresses the function of activated T cells (35–37), and DLBCL patients within the high-risk group may benefit from therapy using PD-L1 inhibitors.

In addition, drug sensitivities for DLBCL patients within different risk groups were further predicted. Our analysis revealed that high-risk patients exhibited a higher IC50 for several drugs, including AKT inhibitor VIII (Inhibitor of AKT kinase), Bleomycin (Inducing DNA damage), Cisplatin (DNA cross-linking agents) and LFM.A13 (Inhibitor of Bruton’s tyrosine kinase), indicating reduced sensitivity to these treatments. Conversely, these patients responded more favorably to drugs such as Etoposide (Topoisomerase II inhibitor), Lenalidomide (Immunomodulator), Rapamycin (mTOR inhibitor) and Vorinostat (Histone deacetylase inhibitor). Thus, our findings suggest a more precise and personalized treatment strategy tailored to the risk profiles of DLBCL patients.

As it is well known, cancer cells exhibit distinct metabolic signatures and utilize much more glucose under adequate nutritional conditions through the Warburg effect to support their rapid proliferation, while they inevitably encounter a nutrient-deprived tumor microenvironment (38). However, cancer cells still maintain proliferation and survival under stressful conditions, in which gluconeogenesis emerges as one of the key factors (39). Gluconeogenesis generates glucose from noncarbohydrate substrates such as lactate and amino acids, and PCK2 is a mitochondrial form of phosphoenolpyruvate carboxykinase (PEPCK or PCK), which catalyzes the first rate-limiting reaction in gluconeogenesis (40). Elevated expression of PCK2 has been observed in several cancer types, including colon cancer, non-small cell lung cancer (NSCLC) and hepatocellular carcinoma (HCC) (38). Conversely, other studies have shown that PCK2 overexpression could suppress the cancer progression in renal cell carcinoma and melanoma (41, 42). This suggests that the role of PCK2 may vary depending on the cancer type and context. The endogenous apoptosis pathway, namely mitochondrial apoptosis, is induced by varying cellular stresses such as growth-factor deprivation, DNA damage or glucose deprivation (43). Previous studies demonstrated that knockdown or inhibition of PCK2 in low glucose or glucose deprivation conditions results in mitochondrial-associated apoptotic cell death in lung cancer (38, 44). In this study, PCK2 was found to be significantly upregulated in DLBCL patients and cell lines. Knockdown of PCK2 in DLBCL cells did not significantly affect cell growth in high glucose conditions; however, it remarkably inhibited cells proliferation and increased apoptosis in low glucose conditions. These findings suggest that PCK2 is a potential therapeutic target for DLBCL.

Our study presents several limitations. Firstly, we utilized DLBCL samples from GEO datasets to establish and validate the prognostic MitoRGs signature; however, this approach lacks confirmation from a larger, real-world cohort. Secondly, since the MitoRGs signature comprises eight genes, we focused solely on the PCK2 gene for our preliminary experimental study. Future research should explore the functional roles of other genes and further investigate PCK2. Despite these limitations, this is the first study to construct a prognostic MitoRGs signature for DLBCL patients, and the risk model provides potential therapeutic targets.

## Conclusion

We developed a novel prognostic MitoRGs signature for the survival of DLBCL patients and conducted preliminary research on the role of PAK2 gene, providing new insights into the prognosis of DLBCL patients and the mechanism of MitoRGs. Future large-scale clinical studies are essential to validate the clinical significance of these findings and to identify more effective treatment strategies for DLBCL.

## Data Availability

The original data in this study is from these public databases and we have previously described them in the manuscript. Other researchers can get information related to our study from these databases: GEO database (https://www.ncbi.nlm.nih.gov/geo/) with accession numbers GSE56315, GSE10846, GSE11318, and GSE87371, MitoCart3.0 database (https://www.broadinstitute.org/mitocarta), GDSC database (https://www.cancerrxgene.org/), GEPIA 2 database (http://gepia2.cancer-pku.cn/#index), HPA database (https://www.proteinatlas.org/).
