# Supplementary material for: Development of a mitochondria-related gene signature for prognostic assessment in diffuse large B cell lymphoma
Source: Front Oncol. 2025 Mar 20;15:1542829. doi: 10.3389/fonc.2025.1542829 (PMC11966244; doi:10.3389/fonc.2025.1542829)
Supplement: Supplementary file 1 [file DataSheet1.docx]

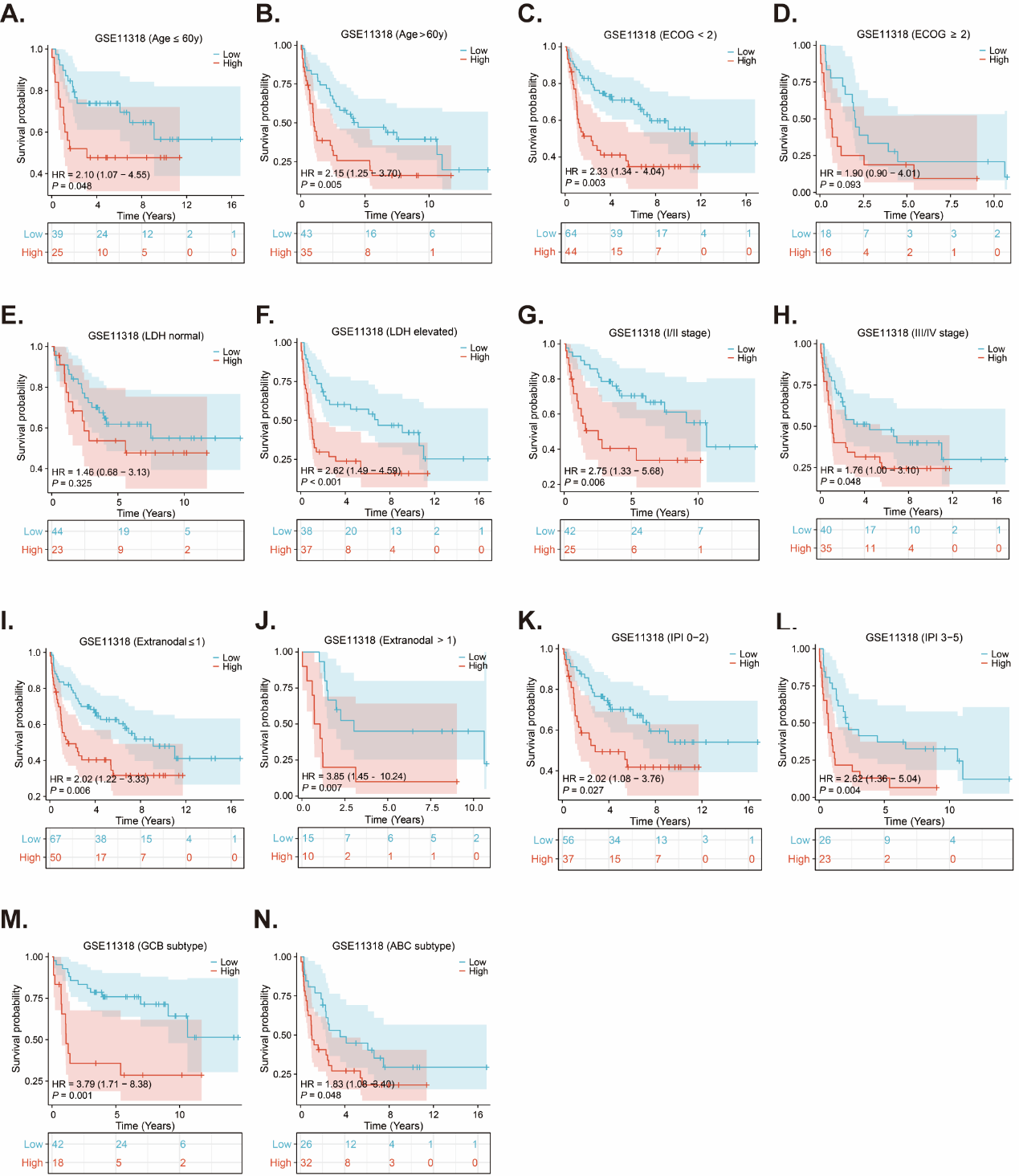


**Figure S1. Survival analyses of the prognostic MitoRGs signature across different subgroups in GSE11318.**

(A-B) The Kaplan-Meier curves of low- and high-risk scores in patients younger than and older than 60 years. (C-D) The Kaplan-Meier curves of low- and high-risk scores in subgroups with ECOG scores less than 2 and those with scores of 2 or more. (E-F) The Kaplan-Meier curves of low- and high-risk scores in subgroups with normal and elevated LDH levels. (G-H) The Kaplan-Meier curves of low- and high-risk scores in subgroups with I/II stage and III/IV stage. (I-J) The Kaplan-Meier curves of low- and high-risk scores in subgroups with one or fewer extra-nodal sites and those with more than one. (K-L) The Kaplan-Meier curves of low- and high-risk scores in subgroups with IPI 0-2 scores and IPI 3-5 scores. (M-N) The Kaplan-Meier curves of low- and high-risk scores in subgroups with GCB and ABC subtypes.

**
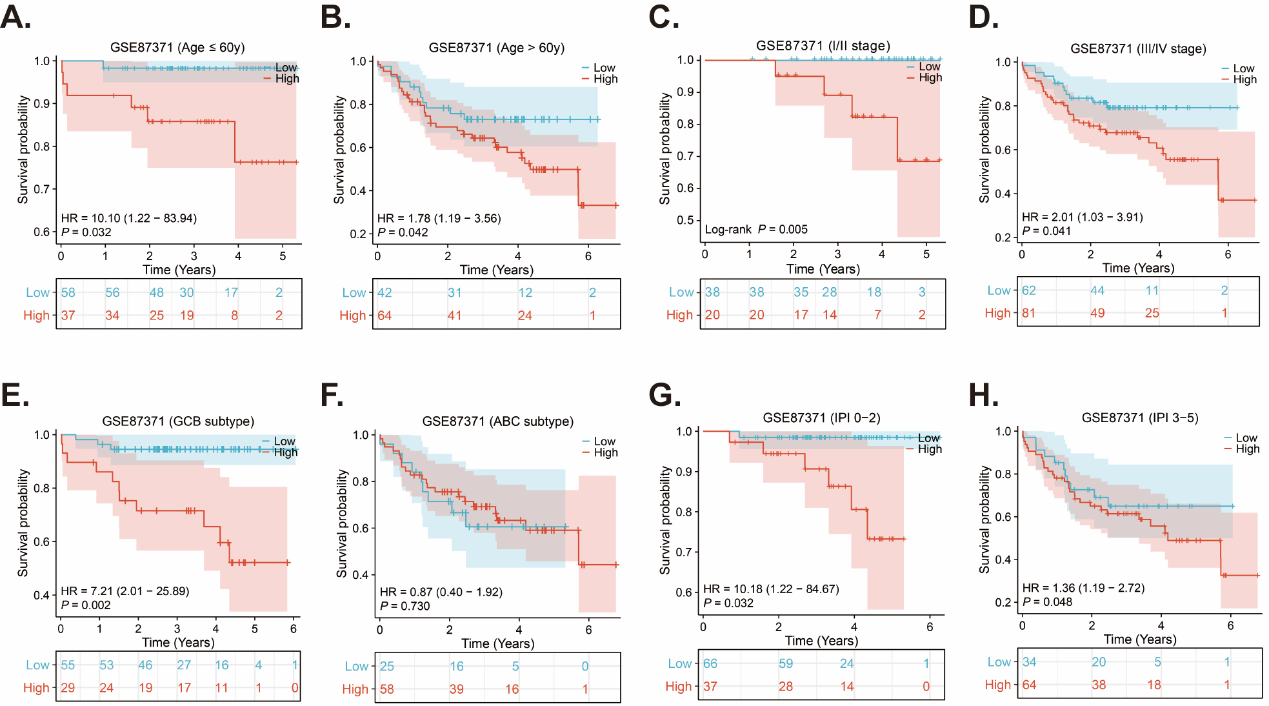
**

**Figure S2. Subgroups survival analyses of the prognostic MitoRGs signature in GSE87371.**

(A-B) The Kaplan-Meier curves of low- and high-risk scores in patients under and over 60 years of age. (C-D) The Kaplan-Meier curves of low- and high-risk scores in subgroups with I/II stage and III/IV stage. (E-F) The Kaplan-Meier curves of low- and high-risk scores in subgroups with GCB and ABC subtypes. (G-H) The Kaplan-Meier curves of low- and high-risk scores in subgroups with IPI 0-2 scores and IPI 3-5 scores.

**
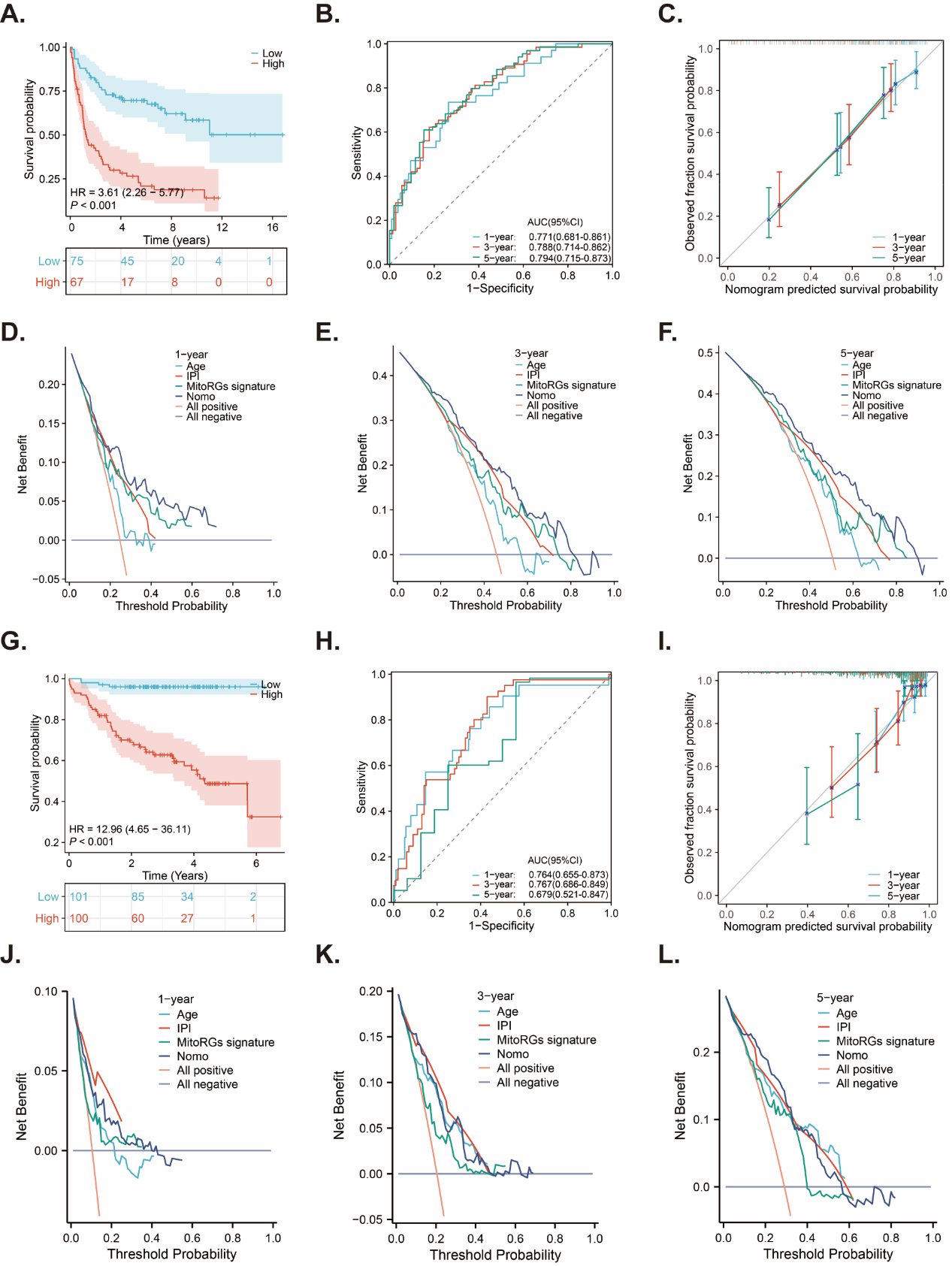
**

**Figure S3. Nomogram model to predict OS of DLBCL patients in validation cohorts.**

(A and G) Kaplan-Meier curves comparing low- and high-risk scores from the nomogram model in GSE11318 and GSE87371, respectively. (B and H) The time-dependent ROC curves of the nomogram model for predicting 1-, 3-, and 5-year OS, along with the corresponding AUC values and 95%CI in GSE11318 and GSE87371, respectively. (C and I) The calibration curves of the nomogram model predicting 1-, 3-, and 5-year OS in GSE11318 and GSE87371, respectively. (D-F and J-L) The Decision curve analysis (DCA) curves of the nomogram model predicting 1-, 3-, and 5-year OS in GSE11318 and GSE87371, respectively.
